# Environmental risk factors for dementia: a systematic review

**DOI:** 10.1186/s12877-016-0342-y

**Published:** 2016-10-12

**Authors:** Lewis O. J. Killin, John M. Starr, Ivy J. Shiue, Tom C. Russ

**Affiliations:** 1 Alzheimer Scotland Dementia Research Centre, University of Edinburgh, Edinburgh, UK; 2 Centre for Cognitive Ageing & Cognitive Epidemiology, University of Edinburgh, Edinburgh, UK; 3 Scottish Dementia Clinical Research Network, NHS Scotland, Perth, UK; 4 Faculty of Health and Life Sciences, Northumbria University, Newcastle-upon-Tyne, UK; 5 Centre for Dementia Prevention, University of Edinburgh, Edinburgh, UK; 6 Division of Psychiatry, Centre for Clinical Brain Sciences, University of Edinburgh, Edinburgh, UK

**Keywords:** Dementia, Alzheimer’s disease, Environment, Epidemiology, Risk factors

## Abstract

**Background:**

Dementia risk reduction is a major and growing public health priority. While certain modifiable risk factors for dementia have been identified, there remains a substantial proportion of unexplained risk. There is evidence that environmental risk factors may explain some of this risk. Thus, we present the first comprehensive systematic review of environmental risk factors for dementia.

**Methods:**

We searched the PubMed and Web of Science databases from their inception to January 2016, bibliographies of review articles, and articles related to publically available environmental data. Articles were included if they examined the association between an environmental risk factor and dementia. Studies with another outcome (for example, cognition), a physiological measure of the exposure, case studies, animal studies, and studies of nutrition were excluded. Data were extracted from individual studies which were, in turn, appraised for methodological quality. The strength and consistency of the overall evidence for each risk factor identified was assessed.

**Results:**

We screened 4784 studies and included 60 in the review. Risk factors were considered in six categories: air quality, toxic heavy metals, other metals, other trace elements, occupational-related exposures, and miscellaneous environmental factors. Few studies took a life course approach. There is at least moderate evidence implicating the following risk factors: air pollution; aluminium; silicon; selenium; pesticides; vitamin D deficiency; and electric and magnetic fields.

**Conclusions:**

Studies varied widely in size and quality and therefore we must be circumspect in our conclusions. Nevertheless, this extensive review suggests that future research could focus on a short list of environmental risk factors for dementia. Furthermore, further robust, longitudinal studies with repeated measures of environmental exposures are required to confirm these associations.

## Background

Dementia is a syndrome of cognitive and functional decline, commonly occurring in later life as a result of neurodegenerative and cerebrovascular processes beginning earlier in the life course [1, 2]. It is a major and growing public health concern with substantial increases projected in the future, particularly in low-to-middle income countries [3–5]. Furthermore, there is now a consensus that a substantial proportion of cases are potentially preventable [6–9]. Preventing or, perhaps more realistically, delaying the clinical onset of dementia would have a substantial effect on disease numbers [10–12]. It has been suggested that approximately a third of Alzheimer’s dementia cases could be attributed to seven potentially modifiable risk factors: diabetes, midlife hypertension and obesity, smoking, depression, cognitive inactivity, and low educational attainment [13, 14]. Adding to this amount of risk explained estimates of population attributable fractions derived from genetic factors [15], it is likely that a large proportion of variance in dementia risk remains unexplained. Therefore, there is an urgent need to identify further potentially modifiable risk factors for dementia.

There is evidence from studying geographical variation in dementia rates that environmental risk factors may also be important in the pathogenesis of dementia [16–19]. Two previous reviews have reported on environmental risk factors for Alzheimer’s disease: both concluded that aluminium in drinking water and electromagnetic fields were important and one also highlighted occupational exposure to solvents and pesticides [20, 21]. However, both these reviews focused on Alzheimer’s disease rather than all-cause dementia and neither of them used robust, systematic review methodology and so may not have covered all potentially important risk factors. Thus, we present the first comprehensive systematic review on environmental risk factors for dementia.

## Methods

### Information sources

We identified studies using three approaches. First, we conducted an electronic search of the PubMed and Web of Science databases from their start to January 2016 (Appendix 1). This search returned relevant primary studies and review articles. Second, we scrutinised bibliographies of included studies and review articles for additional studies which had not been identified by the search. Third, we reviewed a small number of hand-picked studies, based on our knowledge of the literature from scrutiny of articles related to publically available environmental data.

### Search strategy

A shortlist of environmental factors was created based on previous work investigating environmental risk factors on health in general [22]. We also included factors not included in the general list which we knew had been linked to dementia risk, such as trace elements (for example, selenium [23]). Thus, the list of environmental factors which we searched for were air pollution, climate, ultraviolet radiation, green space, industrial pollution, water quality, noise pollution, low-frequency and radiofrequency radiation, radon, nuclear facilities, contamination, and trace elements.

### Screening & eligibility

Records returned from the search were screened based on titles and abstracts for suitability. Conference proceedings, books and editorial publications were excluded. Of the remaining studies, articles were then selected if they reported an association (including null associations) between an environmental factor and any dementia.

The main exclusion criteria were: animal studies; single case studies; studies where the exposure was a physiological measure which could not be directly linked with the environment, for example autopsy studies measuring levels of trace elements in the brain or measuring serum levels of a substance; studies with outcomes other than clinical dementia, e.g. cognitive decline; studies of specific symptoms or features (e.g. inflammation, oxidative stress) of dementia rather than the full dementia syndrome; studies where the exposure was solely nutritional; or where insufficient data were reported in the paper. Since we have recently published a systematic review and meta-analysis on the association between rurality and urbanicity on dementia [16], we did not consider this in the present review. Relevant review studies were carefully examined and additional primary articles not identified in the search were included (see below for definition). However, if a review reported multiple new studies, we included the review as a single source of evidence, summarising its findings rather than including every study individually (for example, Loef and Walach, 2012 [24] included 101 individual studies*).* We refer to the review studies in the main text and, where relevant, performed additional focused searches to identify any more recent articles which would have been included in these reviews. We also reported the most recent report from studies, removing any earlier report(s) (e.g., Rondeau et al., 2000 [25]).

### Quality of evidence

Individual studies were appraised for quality. Large, prospective longitudinal studies with a robust measure of the risk factor in question at baseline and a clinical diagnosis of dementia were classified as being of quality level A. High quality cross-sectional studies (i.e., a large sample size with robust exposure and outcome measures) were considered level B quality, and studies which did not reach this level were considered level C quality. The total evidence for an individual risk factor was synthesised to conclude whether there is strong, moderate, or weak evidence for an association (or lack of an association) between the risk factor and dementia or whether there is insufficient evidence to come to a conclusion. The criteria for each level of strength are given in Table 1. The reporting of this systematic review conforms to the PRIMSA guidelines [26].

**Table 1:** Definitions of the strength of the reported association between individual risk factors and dementia

**Strong evidence** for environmental risk factor	
There is a reported association with dementia in the majority of published papers.	
**Moderate or weak evidence** for environmental risk factor	
Although the reported association with dementia is not seen in the majority of published papers, at least one published paper (or only one) supports the association.	
Weak evidence may come from a single, poorly designed study.	
**No evidence** for environmental risk factor	
No association with dementia has been revealed *either* due to a lack of relevant research *or* the observed effect is not seen as significant.	

## Results

**Fig. 1:**
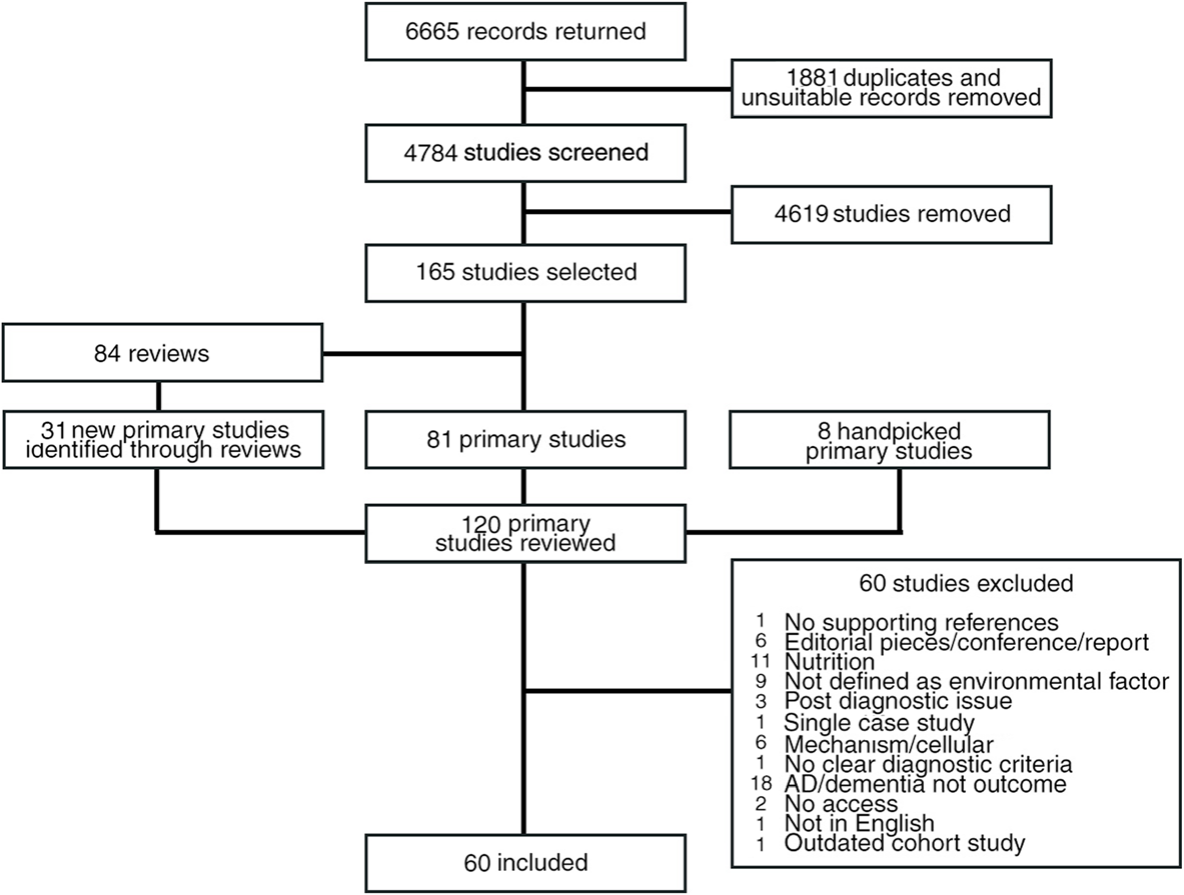
PRISMA diagram showing the selection of studies from search to inclusion

The PRISMA diagram for the process of screening and selecting records is shown in Fig. 1. From an initial total of 6665 studies returned from the searches, after removing duplicates across the environmental factors, 4784 studies were screened for suitability. Of these, 60 were identified as eligible for inclusion.

Table 2 shows the synthesised evidence from all studies with a global judgement about the strength of the evidence for an association from all published studies. The environmental risk factors under consideration fall into the following six groups: air quality, toxic heavy metals, other metals, other trace elements, occupational-related exposures, and miscellaneous environmental factors. Tables 3, 4, 5, 6, 7 and 8 summarise individual studies providing evidence for each risk factor in each category. The tables are organised alphabetically by exposure with higher quality studies being reported first within this grouping and included review studies last of all.

**Table 2:** Quality of overall evidence for each environmental factor

Factor	N Studies^a^			Overall Strength Of Evidence^b^	Direction Of Association^c^
	C	X	R		
Air					
Nitrogen oxides (NO_x_)	2			Strong	↑
Carbon monoxide (CO)	1			Moderate	↑
Environmental tobacco smoke		1		Moderate	↑
Particulate matter (PM_10_&_2.5_)	1	1		Strong	↑
Ozone (O_3_)	1	1		Strong	↑
Toxic heavy metals					
Arsenic		2		Moderate	↕
Lead		1		Weak	↑
Other metals					
Aluminium	1	15		Moderate	↕
Calcium	1			Weak	–
Cobalt		1		Weak	–
Copper		2	1	Weak	↕
Iron		2	1	Weak	↕
Manganese		1		Weak	↑
Molybdenum		1		Weak	–
Nickel		1		Weak	–
Uranium		1		Weak	–
Zinc		2		Weak	↕
Other trace elements					
Fluoride		1		Weak	↑
Selenium			1	Moderate	↕
Silicon (and silica)	2	2		Strong	↕
Occupational					
Aluminium (occupational exposure)	1	3		Weak	↕
Defoliants/fumigants	1			Weak	↑
Diesel motor exhaust	1			Moderate	–
Electromagnetic fields	1			Moderate	↕
Excessive noise	1			Weak	↓
Glues/adhesives	1	1		Weak	↕
Pesticides/fertilizers/herbicides/insecticides	5	2	2	Strong	↕
Lead (occupational exposure)			1	Weak	–
Metals (occupational exposure)	1			Moderate	↑
Inks/dyes	1			Weak	–
Paints/stains/varnishes	1			Weak	–
Gasoline/fuels/oils	1			Weak	–
Solvents/degreasers	2	1	1	Strong	↕
Liquid plastics/rubbers	1			Weak	–
Vibratory tools	1			Weak	–
Radiation	1			Weak	↑
Miscellaneous					
Climate		1		Weak	–
Electric and magnetic fields	1		2	Moderate	↕
Mobile phone use	1			Weak	↓
Vitamin D	3	1		Strong	↑
Water pH		1		Weak	↑

^a^ *C* cohort studies, *X* cross-sectional studies, *R* reviews. Studies can appear in multiple rows

^b^Strength of evidence is assessed according to the criteria outlined in Table 1

^c^Increased levels of the exposure are associated with: ↑ an increased risk of dementia; ↓ a decreased risk of dementia; ↕ mixed results on dementia risk; and – no substantial effect on dementia risk

**Table 3:** Individual studies reporting the association between environmental risk factors in air and dementia

Study	Exposure	Sample description	*N*	Methodology/design	Finding	Grade
Chang et al., 2014 [28]	CO and NO_2_	Comprehensive National Health Insurance database in Taiwan – people aged ≥50 years.	29537 (NO_2_ 29547) men and women of whom 1718 (1720) developed dementia	Retrospective cohort study: Cox PH models. Yearly average CO concentrations based on location of clinic attended. Dementia diagnoses were extracted from electronic health records.	Highest vs lowest quartile of CO and NO_2_ was associated with an increased risk of dementia incidence (multivariable adjusted HR, 95 % CI: 1.54, 1.34-1.77; 1.61, 1.39-1.85). Similar effects in men and women.	A
Oudin et al., 2015 [27]	Traffic-related air pollution (NO_x_)	Participants from the Betula study, randomly sampled from the general population residing in the Umeå municipality	1,806 healthy men and women of whom 302 developed dementia	Prospective cohort study: Cox PH models. Mean nitrogen oxide levels based on baseline residence and dementia outcome after 15 years.	Highest:lowest quartile of NO_x_ revealed an increased risk of incident dementia (adjusted HR, 95 % CI 1.60, 1.02-2.10). Similar results were observed in AD and VaD.	A
Chen et al., 2013 [29]	Environmental Tobacco Smoke	Community dwelling adults aged ≥50 years living in rural or urban areas of five provinces of China.	5921 men and women of whom 626 had severe (O3-5) and 869 moderate (O1-2) dementia	Cross-sectional study. Smoking status and ETS exposure (at home, work, and other places) defined by self-report. Dementia was diagnosed using the GMS-AGECAT algorithm.	Multivariable-adjusted RR (95 % CI) for exposure to ETS 0.96 (0.84, 1.09) for moderate dementia and 1.29 (1.05, 1.59) for severe dementia. Risk of severe dementia increased with increasing duration and cumulative dose, particularly in never smokers.	B
Jung et al. 2015 [33]	PM_2.5_ and O_3_	Individuals from Taiwan entered into the longitudinal health insurance database 2000 (LHID2000) aged ≥65 in 2001.	95,690 men and women of whom 1399 developed AD.	Prospective cohort study: Cox PH models. PM_2.5_ and O_3_ levels recorded at 70 Taiwan Environmental Protection Agency monitoring stations from 2000 to 2010. These data were controlled for secondary pollutants (CO, NO_2_ and SO_2_). AD was identified when this was recorded at least twice on the insurance database, based on a physician diagnosis.	Baseline O_3_ was associated with an increased risk of incident AD (multivariable-adjusted HR per interquartile range, 95 % CI: 1.06, 1.00-1.12) but baseline PM_2.5_ was not (1.03, 0.95-1.11). Change in both O_3_ and PM_2.5_ was associated with increased AD risk (3.12, 2.92-3.33; 2.38, 2.21-2.56). This remained after additionally adjusting for secondary pollutants.	A
Wu et al., 2015 [34]	PM_10_ and O_3_	249 AD patients, 125 VaD patients (clinically diagnosed from hospital clinics) and 497 controls (from the elderly health check-up program), all ≥60 years in Taiwan.	374 cases; 497 controls	Cross-sectional study (case–control): multiple regression. 12-year PM_10_ and 14-year ozone exposure were estimated from spatiotemporal models based on residential location.	Highest vs lowest tertile of PM_10_ and ozone exposure was associated with increased AD risk (adjusted OR, 95 % CI: 4.17, 2.31-7.54; 2.00, 1.14-3.50). Similar results were found for VaD.	B

*AD* = Alzheimer’s dementia, *CO* carbon monoxide, *ETS* environmental tobacco smoke, *GMS-AGECAT* the Automated Geriatric Examination for Computer Assisted Taxonomy algorithm used with the Geriatric Mental State interview (O = “organic” diagnosis from the AGECAT algorithm, levels 1–5), *HR* hazard ratio, *NO*_*2*_ nitrogen dioxide, *O*_*3*_ ozone, *OR* odds ratio, *PH* proportional hazards, *PM*_*x*_ particulate matter up to x micrometres in size, *VaD* vascular dementia

**Table 4:** Individual studies reporting the association between toxic heavy metals and dementia

Study	Exposure	Sample description	*N*	Methodology/design	Finding	Grade
Fox, 2014 [35]	Arsenic	Spring Valley Community, Washington, District of Columbia. Spring Valley was built on a chemical weapons lab which caused arsenic to be distributed to the surface soil.	Population of Spring Valley 2006 – 2010: 24,762	Comparison of annual average age-adjusted mortality rates (per 100,000) between Spring Valley, Chevy Chase (2004 – 2010) and the US (2007). No measure of arsenic concentration is given for these areas at these times.	AD mortality rate in Spring Valley (22.8; CI 20.3 – 25.4) was comparable to the Chevy Chase (22; CI 19 – 25) and US (24.70) rates. No statistically significant difference.	B
Dani, 2010 [36]	Arsenic	Secondary analysis of country-level data.	Country-level data.	Simulation.	Slight increases in arsenic concentration in soil were related to exponential increases in dementia rates at a country level.	C
Emard et al., 1994 [37]	Lead	The IMAGE Project covering the population of Saguenay-Lac-Saint-Jean (SLSJ), Québec.	129 individuals with AD (clinically diagnosed by standard medical services) who were born in SLSJ.	Cross-sectional study: principal components analysis. Samples of aquatic sediment were analysed for geochemical variables. AD cases were identified from a registry.	15 individuals with AD were born in areas with lower than average concentration of lead; 49 were born in areas with higher than average concentrations. This difference was statistically significant (*P* < 0.05).	B

*AD* Alzheimer’s dementia, *CI* confidence interval, *SD* standard deviation, *SEM* standard error of the mean

**Table 5:** Individual studies reporting the association between other metals and dementia

Study	Exposure	Sample description	*N*	Methodology/design	Finding	Grade
Rondeau et al., 2009 [39]	Aluminium	PAQUID: A community-based cohort of 3,777 elderly people aged ≥65 years in SW France.	1925 individuals of whom 461 developed clinically diagnosed dementia (364 AD).	Prospective cohort study: Cox PH models. Mean levels of aluminium in drinking water over the previous decade based on current residential location was linked to incident dementia over 15 years follow up.	Highest:lowest quartile of aluminium in drinking water was associated with an increased risk of dementia and AD (multivariable-adjusted HR, 95 % CI: 2.34, 1.03-5.32; 3.04, 1.32-6.97).	A
Flaten, 1990 [40]	Aluminium	The Norwegian population – mortality data provided by the Central Bureau of Statistics of Norway.	5,642 male and 9,085 female dementia deaths were recorded 1974–1983. The denominator population is estimated at *40.6 million person-years*. The population of Norway was approximately 4 million people during this period.	Cross-sectional study: Pearson’s correlation and relative risk. Four water samples (one per season) were collected in 1982–3 from 384 waterworks and analysed. Small municipalities were aggregated to regions of at least 10,000 inhabitants. Dementia was ascertained from death certification (any mention).	There was a correlation between the aluminium content of drinking water at municipality level and dementia death rates in four time periods from 1969–83 in men and women (*P* < 0.025 and <0.005) but not with PD or ALS death rates. This pattern was less clear at county level.	B
					Highest:lowest tertile of aluminium concentration was associated with an increased risk of dementia death in men and women (RR, 95 % CI: 1.32, *1.20-1.46*; 1.42, *1.32-1.54*).	
Forbes et al., 1995 [41]	Aluminium	Males from the Ontario Longitudinal Study of Aging dying between 1984 and 1991.	3161 patients with AD or young-onset dementia.	Cross-sectional study: Poisson regression. Water quality was based on 30 years’ residential history. Dementia was identified from death certificates (underlying cause).	Highest:lowest tertile of aluminium concentration was associated with an increased risk of AD and AD plus young-onset dementia (RR, 95 % CI: 2.42, 1.42-4.11; 1.96, 1.15-3.32). Effect sizes were greater in analyses restricted to individuals aged ≥75 years.	B
			Total sample not stated.			
					Some models showed a lower risk of dementia in the middle tertile of aluminium concentrations compared to the lowest.	
Neri & Hewitt, 1991 [44]	Aluminium	Patients discharged from general hospitals in Ontario, Canada in 1986.	2344 people aged ≥55 years with a diagnosis of AD or young-onset dementia recorded and 2232 people, matched for age and sex, with a non-psychiatric diagnosis recorded.	Cross-sectional study (case–control). Aluminium concentration in drinking water based on residential location was compared with dementia diagnoses based on hospital discharge statistics.	The authors report a dose–response pattern of association (OR for quartiles 2–4 compared to lowest, 95 % CI: 1.13, *0.55-2.29*; 1.26, *0.61-2.59*; 1.46, *0.71-2.99*) but all CIs (not reported but calculated from data reported in paper) include unity.	B
Gillette-Guyonnet et al., 2005 [57]	Aluminium	Toulouse subset of the EPIDOS study cohort of women aged ≥75 years.	1462 women from one centre of whom 60 developed clinically diagnosed AD. 323 had normal cognitive function and the remainder were lost to follow up.	Prospective cohort study: logistic regression. Water consumption based on self-report (at baseline and follow up) was combined with local tap water composition data. Dementia was clinically diagnosed.	It is not clear if aluminium consumption was included in logistic regression models and not found to be a predictor of dementia or if it wasn’t included.	B
Martyn et al., 1997 [46]	Aluminium	Participants were selected from CT records of eight neuroradiology centres in the UK.	A total of 872 men (106 AD, 99 other dementia, 226 brain cancer, 441 other disease of the nervous system)	Cross-sectional study (case–control): logistic regression. Average levels of aluminium in drinking water based on residential history (after age 25 years) was related to diagnosis based on hospital records.	There were no associations identified between aluminium concentrations over three time periods (from age 25 years to diagnosis; from age 25 years to 10 years before diagnosis; and the ten years preceding diagnosis) with each of the three comparison groups.	B
McLachlan et al., 1996 [43]	Aluminium	Participants were selected from a brain tissue bank in Ontario, Canada.	296 participants had AD, 89 had mixed dementia, 125 had no histopathological abnormalities, and 170 had other conditions (HD, schizophrenia, MS, multiple infarcts, CJD, and other neurodegenerative diseases).	Cross-sectional study (case–control). Average aluminium concentrations in domestic water supply of residential location at death (10 year residential history was available for a subsample) were compared to dementia status (neuropathologically diagnosed).	Aluminium concentration >100 μg/L compared to <100 μg/L was associated with an increased risk of AD and dementia (OR, 95 % CI: 1.7, 1.2-2.6; 1.7, 1.2-2.5).	B
					Effect sizes were larger for the subgroup analysis based on 10-year residential history (2.6, 1.2-5.7; 2.5, 1.2-5.3).	
Frecker, 1991 [42]	Aluminium	Mortality records in Bonavista Bay, Newfoundland, 1985–86.	191 dementia deaths in 1985, 208 deaths in 1986	Cross-sectional study. Place of birth of all individuals dying with dementia was identified and associated with drinking water samples at those locations from 1986.	The area with the highest dementia mortality (37.5 % in 1985 and 68.8 % in 1986) also had the highest aluminium concentrations in drinking water. This association was not assessed for statistical significance, but was argued to not be confounded by age, sex or place of residence stated on death certificate.	B
Emard et al., 1994 [37]	Aluminium	The IMAGE Project covering the population of Saguenay-Lac-Saint-Jean (SLSJ), Québec.	129 individuals with AD (clinically diagnosed by standard medical services) who were born in SLSJ.	Cross-sectional study: principal components analysis. Samples of aquatic sediment were analysed for geochemical variables. AD cases were identified from a registry.	44 individuals with AD were born in areas with lower than average concentration of aluminium; 41 were born in areas with higher than average concentrations. This difference is not statistically significant.	B
Forster et al., 1995 [47]	Aluminium	Patients were drawn from a specialist service in the north of England. Controls were randomly selected from the general population.	109 people with clinically diagnosed young-onset AD and 109 age- and sex-matched controls.	Cross-sectional study (case–control). Aluminium levels in drinking water were determined according to residential history (longest residence in the preceding 10 years plus birthplace for a subset of 80 cases and controls) at local authority district level.	There was no evidence of an association between aluminium concentration in drinking water and dementia risk (e.g. aluminium >149 mg/L OR, 95 % CI: recent residence 1.0, 0.41-2.43; birthplace 1.1, 0.38-3.35).	B
Taylor et al., 1995 [48]	Aluminium	Same cohort as Forster et al., 1995 [47]	Water samples were obtained for 214 addresses of the 218 cases and controls.	Cross-sectional study (case–control). Aluminium concentration in water samples drawn from the place of residence at which they had lived longest within 10 years prior to onset of dementia (or equivalent date for controls).	There were no differences in aluminium concentrations in samples for cases or controls (*P* = 0.60).	B
Gauthier et al., 2000 [107]	Aluminium	Random sample of individuals living in SLSJ aged ≥70 years from the provincial health plan of Quebec in 1994.	68 participants with clinically-diagnosed AD and 68 age- and sex-matched controls.	Cross-sectional study (case–control). Aluminium concentration in drinking water was sampled four times in 1995–6 in 54 municipalities of SLSJ. Long-term exposure was estimated based on residential history.	Of all variables involved in the speciation of aluminium in drinking water (total Al, total dissolved Al, total monomeric Al, organic monomeric Al, inorganic monomeric Al, polymeric Al, as well as the main monomeric inorganic forms) based on recent and long-term exposure, only recent exposure to monomeric organic aluminium was associated with AD at conventional levels (OR, 95 % CI 2.67, 1.04-6.90).	B
Shen et al., 2014 [49]	Aluminium	26 provinces and 3 municipal districts of mainland China.	Not specified.	Cross-sectional study. Soil chemical levels in 1990 were related to AD mortality 1991–2000.	Higher aluminium levels in soil were associated with reduced AD mortality (highest:lowest group RR, 95 % CI: 0.267, 0.265-0.268).	B
Vogt, 1986 [45]	Aluminium	Mortality data from the Central Bureau of Statistics (1969–1983) and dementia patients within 112 psychiatric nursing homes (as collected by Norwegian Institute for Gerontology in 1982).	Not specified.	Cross-sectional study. Drinking water quality and acidification of lakes was related to standardized dementia mortality rates.	Highest:lowest zone of aluminium concentration was associated with a raised standardised dementia mortality rate (per 10,000 inhabitants per year: 48.3 vs 32.4). The effect size was greater in women (59.4 vs 38.5) than men (36.9 vs 26.1).	B
Wettstein et al., 1991 [108]	Aluminium	Residents of two Zurich city districts.	775 men and women aged 82–85 years who had lived in that area for at least 15 years.	Cross-sectional study. Aluminium concentrations were measured in drinking water. The dementia outcome was measured using the truncated MMSE.	No significant difference was observed between the participant groups on mnestic or naming subscores (*P* = 0.962 and *P* = 0.567).	C
Civita, Fiorucci, & Mie, 2001 [109]	Aluminium	AD mortality in municipalities within 20 km of Alba based on national mortality statistics. Census data were used as a reference population.	Not stated.	Cross-sectional study. A map of the location of deaths with AD was compared to levels of aluminium released from 1 kg of clay at various sampling sites.	They found an increased SMR in the municipality compared to the province or the whole of Italy. They also noted that the areas with highest dementia mortality had the highest aluminium concentrations in water.	C
Gillette-Guyonnet et al., 2005 [57]	Calcium	Toulouse subset of the EPIDOS study cohort of women aged ≥75 years.	1462 women from one centre of whom 60 developed clinically diagnosed AD. 323 had normal cognitive function and the remainder were lost to follow up.	Prospective cohort study: logistic regression. Water consumption based on self-report (at baseline and follow up) was combined with local tap water composition data. Dementia was clinically diagnosed.	Women who developed AD showed a decrease in daily calcium intake at follow up. It is not clear if calcium consumption was included in logistic regression models and not found to be a predictor of dementia or if it wasn’t included.	B
Emard et al., 1994 [37]	Cobalt	The IMAGE Project covering the population of Saguenay-Lac-Saint-Jean (SLSJ), Québec.	129 individuals with AD (clinically diagnosed by standard medical services) who were born in SLSJ.	Cross-sectional study: principal components analysis. Samples of aquatic sediment were analysed for geochemical variables. AD cases were identified from a registry.	21 individuals with AD were born in areas with lower than average concentration of cobalt; 20 were born in areas with higher than average concentrations. This difference is not statistically significant.	B
Emard et al., 1994 [37]	Copper	The IMAGE Project covering the population of Saguenay-Lac-Saint-Jean (SLSJ), Québec.	129 individuals with AD (clinically diagnosed by standard medical services) who were born in SLSJ.	Cross-sectional study: principal components analysis. Samples of aquatic sediment were analysed for geochemical variables. AD cases were identified from a registry.	18 individuals with AD were born in areas with lower than average concentration of copper; 30 were born in areas with higher than average concentrations. This difference is not statistically significant.	B
Shen et al., 2014 [49]	Copper	26 provinces and 3 municipal districts of mainland China.	Not specified.	Cross-sectional study. Soil chemical levels in 1990 were related to AD mortality 1991–2000.	Copper concentration correlated with annual AD mortality after three outlier provinces were removed (r = 0.449, *P* = 0.021).	B
Loef & Walach, 2012 [24]	Copper	Systematic review of studies relating copper to AD from 11 databases.	101 studies:	Systematic review.	Of relevant evidence that is reviewed, the authors conclude that “In summary, the current trials provide no conclusive evidence that depletion or supplementation of Cu is beneficial for AD… [t]he specific outcomes for Cu are more conflicting; while evidence suggests that the systemic Cu level is increased in patients with AD, further research is needed to define the alterations of Cu in the brain during AD.” (p.6).	-
			2 meta analyses, 2 systematic reviews, 11 RCTs, 2 prospective studies, 3 cross-sectional studies, 45 case–control studies, 30 autopsy studies,5 uncontrolled studies, 1 case study.			
Emard et al., 1994 [37]	Iron	The IMAGE Project covering the population of Saguenay-Lac-Saint-Jean (SLSJ), Québec.	129 individuals with AD (clinically diagnosed by standard medical services) who were born in SLSJ.	Cross-sectional study: principal components analysis. Samples of aquatic sediment were analysed for geochemical variables. AD cases were identified from a registry.	16 individuals with AD were born in areas with lower than average concentration of iron; 35 were born in areas with higher than average concentrations. This difference was statistically significant (*P* < 0.05).	B
Shen et al., 2014 [49]	Iron	26 provinces and 3 municipal districts of mainland China.	Not specified.	Cross-sectional study. Soil chemical levels in 1990 were related to AD mortality 1991–2000.	Higher iron levels in soil were associated with increased AD mortality (highest:lowest group RR, 95 % CI: 1.248, 1.245-1.251). Iron concentration correlated with annual AD mortality after three outlier provinces were removed (r = 0.537, *P* = 0.007).	B
Loef & Walach, 2012 [24]	Iron	Systematic review of studies relating iron to AD from 11 databases.	101 studies:	Systematic review.	“In summary, the current trials provide no conclusive evidence that depletion or supplementation of … Fe is beneficial for AD… Fe has been consistently found at elevated levels in the brains of AD sufferers by both autopsy and case–control studies.” (p.6).	-
			2 meta analyses, 2 systematic reviews, 11 RCTs, 2 prospective studies, 3 cross-sectional studies, 45 case–control studies, 30 autopsy studies,5 uncontrolled studies, 1 case study.			
Emard et al., 1994 [37]	Manganese	The IMAGE Project covering the population of Saguenay-Lac-Saint-Jean (SLSJ), Québec.	129 individuals with AD (clinically diagnosed by standard medical services) who were born in SLSJ.	Cross-sectional study: principal components analysis. Samples of aquatic sediment were analysed for geochemical variables. AD cases were identified from a registry.	12 individuals with AD were born in areas with lower than average concentration of manganese; 35 were born in areas with higher than average concentrations. This difference was statistically significant (*P* < 0.05).	B
Emard et al., 1994 [37]	Molybdenum	The IMAGE Project covering the population of Saguenay-Lac-Saint-Jean (SLSJ), Québec.	129 individuals with AD (clinically diagnosed by standard medical services) who were born in SLSJ.	Cross-sectional study: principal components analysis. Samples of aquatic sediment were analysed for geochemical variables. AD cases were identified from a registry.	29 individuals with AD were born in areas with lower than average concentration of molybdenum; 16 were born in areas with higher than average concentrations. This difference is not statistically significant.	B
Emard et al., 1994 [37]	Nickel	The IMAGE Project covering the population of Saguenay-Lac-Saint-Jean (SLSJ), Québec.	129 individuals with AD (clinically diagnosed by standard medical services) who were born in SLSJ.	Cross-sectional study: principal components analysis. Samples of aquatic sediment were analysed for geochemical variables. AD cases were identified from a registry.	27 individuals with AD were born in areas with lower than average concentration of nickel; 29 were born in areas with higher than average concentrations. This difference is not statistically significant.	B
Emard et al., 1994 [37]	Uranium	The IMAGE Project covering the population of Saguenay-Lac-Saint-Jean (SLSJ), Québec.	129 individuals with AD (clinically diagnosed by standard medical services) who were born in SLSJ.	Cross-sectional study: principal components analysis. Samples of aquatic sediment were analysed for geochemical variables. AD cases were identified from a registry.	10 individuals with AD were born in areas with lower than average concentration of uranium; 9 were born in areas with higher than average concentrations. This difference is not statistically significant.	B
Emard et al., 1994 [37]	Zinc	The IMAGE Project covering the population of Saguenay-Lac-Saint-Jean (SLSJ), Québec.	129 individuals with AD (clinically diagnosed by standard medical services) who were born in SLSJ.	Cross-sectional study: principal components analysis. Samples of aquatic sediment were analysed for geochemical variables. AD cases were identified from a registry.	29 individuals with AD were born in areas with lower than average concentration of aluminium; 30 were born in areas with higher than average concentrations. This difference is not statistically significant.	B
Shen et al., 2014 [49]	Zinc	26 provinces and 3 municipal districts of mainland China.	Not specified.	Cross-sectional study. Soil chemical levels in 1990 were related to AD mortality 1991–2000.	Higher zinc levels in soil were associated with increased AD mortality (highest:lowest group RR, 95 % CI: 2.289, 2.275-2.303).	B

*AD* Alzheimer’s dementia, *ALS* amyotrophic lateral sclerosis, *CI* confidence interval, *CJD* Creutzfeld-Jakob Disease, *CT* computed tomography, *HD* Huntington’s disease, *HR* hazard ratio, *MCI* mild cognitive impairment, *MMSE* mini-mental state examination, *MS* multiple sclerosis, *PD* Parkinson’s disease, *PH* proportional hazards, *RCT* randomized, controlled trial, *RR* relative risk, *SD* standard deviation, *SEM* standard error of the mean, *SMR* Standardised Mortality Ratio

Figures in *italics* indicate data not reported but which have been calculated from data in the paper

**Table 6:** Individual studies reporting the association between other trace elements and dementia

Study	Exposure	Sample description	*N*	Methodology/design	Finding	Grade
Still & Kelley, 1980 [51]	Fluoride	All first admissions of people aged ≥55 years to South Carolina Department of Mental Health hospitals from three counties who had lived in that county for at least 10 years.	Case records for 160 patients were examined and, based on that evidence, 67 were diagnosed with a primary degenerative dementia, i.e. AD.	Cross-sectional study. Annual incidence of dementia was calculated per county, based on the admissions data. These were then compared with fluoride concentrations in public water supplies (time of measurement is not given).	Horry County was reported to have highest levels of fluoride (4.18 ± 0.19 mg/l) compared to Anderson (0.49 ± 0.10 mg/l) and York (0.61 ± 0.12 mg/l) Counties. It also has the lowest calculated annual incidence of dementia per 100,000 population (3.6; Anderson 20.8, York 17.1).	C
Loef, Schrauzer, & Walach, 2011 [23]	Selenium	Systematic review of studies relating selenium to AD from 8 databases.	56 studies: 9 placebo-controlled, 4 prospective, 4 cross-sectional, 15 case–control, and 24 autopsy studies.	Systematic review.	One double-blind RCT is reported (PREADVISE[52–54]) but this seems to have subsequently converted into an observational study.	-
					A prospective cohort study is referred to by citing a conference abstract [55]; subsequent reports do not focus on selenium.[56]	
					Of 15 case–control studies, the authors comment that “four studies reported about increased levels of Se-concentration or GPx-activity while the majority found decreased levels, albeit non-significant in some studies.” (p. 87).	
					Of the 24 autopsy studies, the authors conclude that “the autopsy studies do not yield a consistent picture of whether, how and where in brain Se levels become altered in subjects with AD.” (p. 87).	
Gillette-Guyonnet et al., 2005 [57]	Silica	Toulouse subset of the EPIDOS study cohort of women aged ≥75 years.	1462 women from one centre of whom 60 developed clinically diagnosed AD. 323 had normal cognitive function and the remainder were lost to follow up.	Prospective cohort study: logistic regression. Water consumption based on self-report (at baseline and follow up) was combined with local tap water composition data. Dementia was clinically diagnosed.	Low silica consumption in water (≤4 mg vs >12 mg per day) was associated with an increased risk of dementia (multivariable-adjusted OR, 95 % CI: 2.74, 1.09-6.86). Women who developed AD had lower silica intake at baseline and showed a decrease in daily silica intake at follow up.	A
Rondeau et al., 2009 [39]	Silica	PAQUID: A community-based cohort of 3,777 elderly people aged ≥65 years in SW France.	1925 individuals of whom 461 developed clinically diagnosed dementia (364 AD).	Prospective cohort study: Cox PH models. Mean levels of silica in drinking water over the previous decade based on current residential location was linked to incident dementia over 15 years follow up.	Highest:lowest quartile of silica in drinking water was associated with an increased risk of dementia and AD (multivariable-adjusted HR, 95 % CI: 1.33, 1.01-1.74; 1.33, 0.98-1.80).	A
Taylor et al., 1995 [48]	Silica	Same cohort as Forster et al., 1995 [47]	Water samples were obtained for 214 addresses of the 218 cases and controls.	Cross-sectional study (case–control). Aluminium concentration in water samples drawn from the place of residence at which they had lived longest within 10 years prior to onset of dementia (or equivalent date for controls).	There were no differences in silica concentrations in samples for cases or controls and no association between increased levels of silica and dementia (OR, 95%CI ≥3 mg/L 0.8, 0.34-1.83).	B
Martyn et al., 1997 [46]	Silicon	Participants were selected from CT records of eight neuroradiology centres in the UK.	A total of 872 men (106 AD, 99 other dementia, 226 brain cancer, 441 other disease of the nervous system)	Cross-sectional study (case–control): logistic regression. Average levels of silicon in drinking water based on residential history (after age 25 years) was related to diagnosis based on hospital records.	There were no associations identified between silicon concentrations consistently above 6 mg/L (compared to lower levels) with each of the three comparison groups (e.g. AD vs other diagnoses (adjusted-OR, 95 % CI: 0.94, 0.39-2.26).	B

*AD* Alzheimer’s dementia, *CI* confidence interval, *CT* computed tomography, *HR* hazard ratio, *OR* odds ratio, *PH* proportional hazards, *RCT* randomized, controlled trial, *RR* relative risk, *SD* standard deviation, *SMR* Standardised Mortality Ratio, *UK* United Kingdom

**Table 7:** Individual studies reporting the association between occupational exposures and dementia

Study	Exposure	Sample description	*N*	Methodology/design	Finding	Grade
Peters et al., 2013 [60]	Aluminium (occupational exposure)	Survey information collected from miners living in Kalgoorlie, Western Australia. Data collected in 1961,’62,’74,’75 and 2000.	1894 ever underground gold miners linked with Western Australian Registrar General’s Mortality Database of whom 16 died with AD.	Retrospective cohort study: SMRs and Cox PH models. Exposure to aluminium dust ascertained by self-report.	Aluminium dust inhalation was associated with increased AD mortality (SMR, 95 % CI: 1.38, 0.69-2.75). There was also an association between inhaled aluminium dust and AD mortality in Cox models (HR, 95 % CI: 2.76, 0.88-8.82).	B
Salib et al., 1996 [61]	Aluminium (occupational exposure)	Individuals referred to a psychogeriatric unit in Warrington, UK	198 AD, 194 other dementia, 176 unmatched controls.	Cross-sectional study (case–control). Occupation based on self-report.	No association was found between working in the aluminium industry and AD (OR, 95 % CI: 0.98, 0.53-1.75). This was also the case for all dementias	B
Graves et al., 1998 [58]	Aluminium (occupational exposure)	Subsample of Kukull et al.’s cohort [71].	89 AD and 89 matched controls.	Cross-sectional study (case–control). Aluminium exposure rated blind based on occupational history.	There was an association between ever exposure to aluminium and AD (OR, 95 % CI: 1.46, 0.62-3.42) but not in models which took into account intensity, duration, or age at exposure.	B
Gun et al., 1997 [59]	Aluminium (occupational exposure)	Men and women recruited from Sydney hospitals 1986–1989.	170 probable or possible AD and 170 controls.	Cross-sectional study (case–control). Aluminium exposure was derived from an occupational interview.	Aluminium exposure was associated with a reduced odds ratio of AD (OR, 95 % CI: 0.33, 0.01-4.16). This study has very low statistical power because only four cases and controls were exposed. Probable and Possible AD cases considered together.	C
Tyas et al., 2001 [62]	Defoliants/fumigants	The Manitoba Study of Health and Aging – random sample from provincial health insurance records.	694 cognitively-intact older adults followed up for five years, during which time 36 developed probable or possible AD (clinically diagnosed).	Prospective longitudinal study. Exposures based on self-report.	Exposure to defoliants/fumigants was associated with an increased risk of AD (multivariable-adjusted RR, 95 % CI: 4.35, 1.05-17.90).	A
Koeman et al. 2015 [63]	Diesel motor exhaust (DME)	The Netherlands Cohort Study which consisted of 120,852 individuals aged 55–69 years in 1986.	682 men and 870 women who had died with non-VaD reported on their death certificates over 17 years follow up.	Case-cohort study: Cox PH models. Exposures based on self-report. Dementia status ascertained using death certification. Person-years were calculated from a randomly-selected sub-cohort.	Exposure to DME compared to no exposure was not associated with an increased risk of non-VaD mortality in men or women.	A
Koeman et al. 2015 [63]	Electromagnetic fields (ELF-MF and electric shocks)	The Netherlands Cohort Study which consisted of 120,852 individuals aged 55–69 years in 1986.	682 men and 870 women who had died with non-VaD reported on their death certificates over 17 years follow up.	Case-cohort study: Cox PH models. Exposures based on self-report. Dementia status ascertained using death certification. Person-years at risk were calculated from a randomly-selected sub-cohort.	Low or high exposure to ELF-MF compared to no exposure was not associated with an increased risk of non-VaD mortality in men (adjusted HR, 95 % CI 1.26, 1.01-1.57; 1.40, 0.92-2.14) but not in women. Conversely, an association was seen for electrical shocks for women (1.25, 0.85-1.84; 11.1, 3.84-32.2) but not men.	A
					The hazard associated with cumulative ELF-MF exposure to showed no trend (*P* = 0.09).	
Tyas et al., 2001 [62]	Excessive noise	The Manitoba Study of Health and Aging – random sample from provincial health insurance records.	694 cognitively-intact older adults followed up for five years, during which time 36 developed probable or possible AD (clinically diagnosed).	Prospective longitudinal study. Exposures based on self-report.	Occupational exposure to excessive noise was associated with a decreased risk of AD (multivariable-adjusted RR, 95 % CI: 0.12, 0.02-0.96). N.B. only one case was exposed to excessive noise.	B
McDowell et al., 1994 [66]	Glues and pesticides/fertilizers	Canadian Study of Health and Aging based in 10 provinces.	258 people clinically diagnosed with probable AD (less than three years since onset of symptoms) and 535 age-matched controls (stratified by study centre, community-/institution-dweller and clinically confirmed to be cognitively normal)	Cross-sectional study (case–control): logistic regression. Risk factor exposure gathered by questionnaire self-report.	Occupational exposure to glues and pesticides/fertilizers was associated with an increased risk of AD (multivariable-adjusted OR, 95%CI: glues 1.80, 0.99-3.27; pesticides/fertilizers 1.58, 0.81-3.10. Stratifying by education showed higher risk in those with less education.	B
Gauthier et al., 2001 [64]	Herbicides, insecticides and pesticides	1924 people aged ≥70 years old were screened and examined in the Saguenay–Lac Saint-Jean region (Québec, Canada).	122 people clinically diagnosed with AD and 122 age-matched controls (±2 years). 67 case–control pairs hade complete data and were included in the models.	Cross-sectional study (case–control): logistic regression. Exposure to pesticides derived from residential history and census data (1971–91) on herbicide and insecticide spraying in the area.	No increased risk of AD with exposure to herbicides, insecticides or pesticides (multivariable adjusted OR, 95 % CI: 1.07, 0.39-2.54); 1.62 (0.64-4.11); 0.97 (0.38-2.41).	B
Santibanez et al., 2007 [68]	Lead (occupational exposure)	Systematic review of studies linking occupational exposures and AD.	Twenty four studies: 3 cohort and 21 case–control studies.	Systematic review.	“For lead exposure there are no data supporting any association. All the studies are case–control studies, with a relatively low level of quality according to our classification.” (p. 730)	-
Koeman et al. 2015 [63]	Metals (occupational exposure)	The Netherlands Cohort Study which consisted of 120,852 individuals aged 55–69 years in 1986.	682 men and 870 women who had died with non-VaD reported on their death certificates over 17 years follow up.	Case-cohort study: Cox PH models. Exposures based on self-report. Dementia status ascertained using death certification. Person-years at risk were calculated from a randomly-selected sub-cohort.	Low or high exposure to metals compared to no exposure was associated with an increased risk of non-VaD mortality in men (adjusted HR, 95 % CI 1.21, 0.84-1.74; 1.35, 0.98-1.86) and women (4.55, 1.35-15.3; 1.78, 0.23-13.8).	A
					The hazard associated with cumulative exposure to metals showed a significant trend (*P* = 0.01).	
Hayden et al., 2010 [65]	Pesticides	The Cache County study (Utah, USA).	3,084 individuals aged ≥65 years without dementia followed up over 10 years during which time 500 people developed clinically-diagnosed dementia (344 AD).	Prospective cohort study: Cox PH models. Exposures based on self-report. Follow-up conducted at 3, 7 and 10 years from baseline.	Pesticide exposure was associated with an increased risk of dementia (adjusted HR, 95 % CI 1.38, 1.09-1.76) and AD (1.42, 1.06-1.91). A slightly greater risk associated with organophosphates for AD was reported.	A
Baldi et al. 2003 [70]	Pesticides	PAQUID cohort [110]. Male and female residents of Gironde, France aged ≥65 in 1987.	96 incident cases of AD from 1,507 contactable individuals between 5- and 10-year follow-up.	Prospective cohort study.	Occupational exposure to pesticides was associated with an increased risk of AD in men (adjusted RR, 95 % CI: 2.4, 1.0-5.6). This was not the case for other pesticide variables or in women.	A
				Cumulative exposure to pesticides was calculated based on job history. AD was diagnosed by a neurologist.		
Koeman et al. 2015 [63]	Pesticides	The Netherlands Cohort Study which consisted of 120,852 individuals aged 55–69 years in 1986.	682 men and 870 women who had died with non-VaD reported on their death certificates over 17 years follow up.	Case-cohort study: Cox PH models. Exposure based on self-report. Dementia status ascertained using death certification. Person-years at risk were calculated from a randomly-selected sub-cohort.	Exposure to any pesticides was not associated with an increased risk of non-VaD mortality in men or women. The same pattern was seen when disaggregating the effect of insecticides, herbicides, and fungicides in men. In women, high exposure to herbicides and fungicides was associated with an increased risk of non-VaD mortality (adjusted HR, 95 % CI 5.27, 1.30-21.4; 2.83, 0.87-9.16).	A
					The hazard associated with cumulative exposure showed a significant trend for all pesticides and individual types (*P* = 0.01) but the trend was reversed with increasing exposure associated with a lower risk.	
Povey et al., 2014 [67]	Pesticides	British farmers in the 1970s.	1,350 individuals born before 1958.	Retrospective cohort study: logistic regression. Exposure was based on self-reported levels of organophosphate exposure. Low dose chronic exposure was defined as handling organophosphate concentrate and acute exposure as having sought advice for pesticide poisoning. Dementia was identified by a screening questionnaire.	In people who had never sought advice for pesticide poisoning, handling pesticide concentrate was not associated with dementia. However, in those who had handled pesticide concentrate, seeking advice for pesticide poisoning was associated with an increased risk of dementia (adjusted OR, 95 % CI 4.27, 1.85-9.83).	B
Zaganas et al. 2013 [69]	Pesticides	Review of studies linking pesticide exposure and dementia.	Fourteen studies are reviewed (2 on cognitive performance, 1 mild cognitive dysfunction, 7 AD, 1 FTD, 2 VaD, and 1 PD).	Narrative review.	Of the 7 AD studies, 5 demonstrated an increased risk of AD (plus one which did so weakly) and one showed no association.	-
					“Information from the literature [on VaD] is scant; however, occupational exposure to pesticides or fertilizers conferred a two-fold increased risk of developing vascular dementia in the Canadian Study of Health and Aging.” (p. 7).	
					“Due to the relative rarity of [FTD] at the population level, pesticide exposure has been studied as a contributing factor to FTD onset in relatively few studies and no association was found.” (p. 7).	
Santibanez et al., 2007 [68]	Pesticides	Systematic review of studies linking occupational exposures and AD.	Twenty four studies: 3 cohort and 21 case–control studies.	Systematic review.	“For pesticides, research of greater quality and prospective design found increased and statistically significant associations with AD. … The two case control studies assessing risk associated with pesticide exposure and with GQI above the median found evidence of smaller and non-significant associations, supporting the hypothesis that potential biases might have affected these results, decreasing the associations towards the null.” (p. 729–31)	-
Tyas et al., 2001 [62]	Pesticides/fertilizers, inks/dyes, paints/stains/varnishes, gasoline/fuels/oils, solvents/degreasers, liquid plastics/rubbers, glues/adhesives, vibratory tools	The Manitoba Study of Health and Aging – random sample from provincial health insurance records.	694 cognitively-intact older adults followed up for five years, during which time 36 developed probable or possible AD (clinically diagnosed).	Prospective longitudinal study. Exposures based on self-report.	Other occupational exposures were not associated with an increased risk of AD (multivariable-adjusted RR, 95 % CI: pesticides/fertilizers 1.45, 0.57-3.68; inks/dyes 0.89, 0.28-2.81; paints/stains/varnishes 1.21, 0.46-3.21; gasoline/fuels/oils 0.79, 0.29-2.20; solvents/degreasers 0.88, 0.31-2.50; liquid plastics/rubbers 1.01, 0.12-8.38; glues/adhesives 1.41, 0.49-4.05; use of vibratory tools 1.03, 0.20-5.29).	A
Tyas et al., 2001 [62]	Radiation	The Manitoba Study of Health and Aging – random sample from provincial health insurance records.	694 cognitively-intact older adults followed up for five years, during which time 36 developed probable or possible AD (clinically diagnosed).	Prospective longitudinal study. Exposures based on self-report.	Occupational exposure to radiation was associated with an increased risk of AD (multivariable-adjusted RR, 95 % CI: 3.57, 0.38-33.38). N.B. only one case was exposed to radiation.	B
Kukull et al. 1995 [71]	Solvents	Individuals aged ≥60 years recruited to the Group Health Cooperative in Seattle-area clinics.	193 cases of AD and 243 unmatched controls.	Cross-sectional study (case–control): logistic regression. Risk factor exposure was based on self-report.	Solvent exposure was associated with an increased odds ratio of dementia in men (adjusted OR, 95 % CI: 6.3, 2.2-18.1) but not in women (0.6, 0.2-1.9). In men, a significant effect of exposure duration was observed.	B
Koeman et al. 2015 [63]	Solvents	The Netherlands Cohort Study which consisted of 120,852 individuals aged 55–69 years in 1986.	682 men and 870 women who had died with non-VaD reported on their death certificates over 17 years follow up.	Case-cohort study: Cox PH models. Exposures based on self-report. Dementia status ascertained using death certification. Person-years at risk were calculated from a randomly-selected sub-cohort.	High exposure to any solvents compared to no exposure was associated with an increased risk of non-VaD mortality in men (adjusted HR, 95 % CI 1.20, 0.90-1.61) but not women. High exposure to aromatic solvents was associated with increased non-VaD mortality in women (3.46, 0.59-20.5) but not men. High exposure to chlorinated solvents was associated with increased risk in men (1.33, 0.96-1.83) and women (2.08, 0.60-7.14), as was low exposure in men (1.25, 0.89-1.76).	A
					The hazard associated with cumulative exposure to chlorinated solvents showed a significant trend (*P* = 0.01).	
Santibanez et al., 2007 [68]	Solvents	Systematic review of studies linking occupational exposures and AD.	Twenty four studies: 3 cohort and 21 case–control studies.	Systematic review.	“For solvents, only two out of the 11 studies analysing this exposure found a significant association with AD.” (p. 730)	-

*AD* Alzheimer’s disease, *CI* confidence interval, *ELF-MF* Extremely low frequency magnetic fields, *FTD* fronto-temporal dementia, *HR* hazard ratio; *OR* odds ratio, *PD* Parkinson’s disease, *RR* relative risk, *VaD* vascular dementia

**Table 8:** Individual studies reporting the association between miscellaneous exposures and dementia

Study	Exposure	Sample description	*N*	Methodology/design	Finding	Grade
Salib & Sharp, 1999 [72]	Climate	Daily hospital admissions from North Cheshire to a single hospital. Dementia cases were identified by ICD-9 code 290.	189/2070 psychiatric admissions during 1993 were coded as being related to dementia.	UK Meteorological Office data were collected at Manchester airport (30 miles away) every day.	There were no associations found between weather parameters and hospital admissions of people with dementia.	C
Huss et al., 2009 [74]	Magnetic field exposure (220–380 kV) through power lines	Swiss National Cohort from 2000–5. The study population comprised 4.65 million individuals and 22,821,824 person-years.	29,975 dementia deaths were recorded, including 9,228 AD deaths.	Prospective cohort study: Cox PH models. Exposure was based on distance of place of residence to the nearest power line and duration of exposure (5, 10, or 15 years). Dementia was ascertained from death certification.	Proximity to power lines was associated with an increased risk of dementia but this was not statistically significant at conventional levels (adjusted HR, 95 % CI closest:most distant categories 1.23, 0.96-1.59). Longer duration increased the magnitude of this association (≥15 years at this place of residence 2.00, 1.21-3.33).	A
Vergara et al., 2013 [111]	Extremely low frequency magnetic fields	Systematic review of studies of occupational exposure to magnetic fields and neurodegenerative disease.	20 AD and 9 dementia studies.	Systematic review and meta-analysis.	There was a small association between occupational magnetic field exposure and AD based on a meta-analysis (RR, 95 % CI 1.27, 1.15-1.40). There was no clear association with dementia (1.05, 0.96-1.14).	-
Garcia et al., 2008 72]	Extremely low frequency electric and magnetic fields.	Systematic review and meta-analysis	Fourteen studies: 9 case–control, 5 cohort	Systematic review.	Pooled cohort risk estimates (OR, 95 % CI 1.62, 1.16-2.27). Pooled case–control risk estimates (2.03, 1.38-3.00, *P* = 0.004).	-
Schuez et al., 2009 [75]	Mobile phones	All mobile phone subscriptions in Denmark, 1982–1995.	420,095 private mobile phone subscribers of whom 532 were admitted to hospital with a dementia code during follow up.	Prospective cohort study. Mobile phone use was derived from subscription records. Dementia status was identified from hospital admission records.	Mobile phone use was associated with a decreased risk of being hospitalised with AD (Standardized hospitalization ratio, 95 % CI 0.7, 0.6-0.9). Similar results were seen for VaD and “other dementia”.	B
Afzal et al., 2014 [76]	Vitamin D	Danish general population sample recruited to the Copenhagen Heart Study at baseline (1981 to 1983).	10,186 participants, of whom 418 developed AD and 92 developed to VaD.	Prospective cohort study: Cox PH models. Baseline plasma vitamin D levels were related to incident AD and VaD. Dementia status was derived from diagnostic codes recorded on the national Danish Patient Registry.	Lower plasma vitamin D levels were associated with an increased risk of AD (HR, 95 % CI <25^th^ percentile [seasonally-adjusted] vs >50^th^ 1.29, 1.01-1.66; *P* = 0.03). Similar findings were reported for VaD (1.22, 0.79-1.87; *P* = 0.42) and all dementia (1.27, 1.01-1.60; *P* = 0.02).	A
Littlejohns et al., 2014 [78]	Vitamin D	Healthy participants in the US population–based Cardiovascular	1658 adults followed up for mean 5.6 years during which time 171 developed clinically-ascertained dementia (102 AD).	Prospective cohort study: Cox PH models. Serum vitamin D levels were measured at baseline.	Being deficient (25-50nM) or severely deficient (<25nM) in vitamin D was associated with an increased risk of incident AD (multivariable-adjusted HR, 95 % CI: 1.69, 1.06-2.69; 2.22, 1.02-4.83; P_trend_ = 0.008). Similar results were seen for all-cause dementia (1.53, 1.06-2.21; 2.25, 1.23-4.13; P_trend_ = 0.002).	A
		Health Study.				
Annweiler et al., 2011 [77]	Vitamin D	Toulouse subset of the EPIDOS study cohort of women aged ≥75 years.	40 participants, of whom 6 developed non-AD (and 4 AD) at clinical assessment over 7 years follow up.	Prospective cohort study: logistic regression. Baseline serum vitamin D was related to incident non-AD dementia.	Vitamin D deficiency at baseline was associated with an increased risk of non-AD dementia (adjusted OR, 95 % CI 19.57, 1.11-343.69; *P* = 0.042).	A
Wilkins et al., 2006 [79]	Vitamin D	Participants recruited from greater metropolitan St Louis, MO by the Washington University AD Research Center.	40 people with clinically diagnosed mild AD and 40 without dementia.	Cross-sectional study (case–control): logistic regression and general linear model. Serum vitamin D levels were measured.	Vitamin D status was not associated with AD (multivariable-adjusted OR, 95 % CI: deficient vs sufficient 2.80, 0.64-12.28; insufficient vs sufficient 1.78, 0.61-5.19). Vitamin D status was associated with CDR sum of boxes (*P* = 0.0468); and the Short Blessed Test (*P* = 0.0077) but not others tests.	B
Frecker, 1991 [42]	Water pH	Mortality records in Bonavista Bay, Newfoundland, 1985–86.	191 dementia deaths in 1985, 208 deaths in 1986	Cross-sectional study. Place of birth of all individuals dying with dementia was identified and associated with drinking water samples at those locations from 1986.	The area with the highest dementia mortality (37.5 % in 1985 and 68.8 % in 1986) also had the lowest pH of drinking water (5.2). This association was not assessed for statistical significance, but was argued to not be confounded by age, sex or place of residence stated on death certificate.	B

*AD* Alzheimer’s disease, *CI* confidence interval, *HR* hazard ratio, *OR* odds ratio, *PH* proportional hazards, *UK* United Kingdom, *VaD* vascular dementia

### Air quality

Studies of the association between environmental risk factors in air and dementia are summarised in Table 3. Higher levels of **nitrogen oxides** were observed to be associated with increased dementia risk in two prospective cohort studies: a 15 year prospective cohort study of 1806 healthy men and women in Umeå, Sweden, and a retrospective cohort study of almost thirty thousand individuals identified from the comprehensive Taiwanese health insurance database passively followed up for a decade [27, 28]. However, only the prospective study observed a dose–response pattern of association (adjusted HR per 10 μg/m^3^ increase in nitrogen oxides, 95 % CI 1.05, 0.98, 1.12) [27]. This study grouped nitrogen oxides into the following cut-offs: 9, 17, and 26 μg/m^3^. The other study only observed an increased risk in the highest quartile and used the following cut-offs for NO_2_ concentration: 6652.3, 8349.0, and 9825.5 parts per billion [28]. The discrepancy in the pattern of observation observed in these two studies can be explained by the fact that the former explored higher levels of NO_x_ – the highest cut-off from the latter study (~10 parts per million) approximately corresponds to the lowest cut-off from the former study (~10 mg/L ≡ μg/m^3^). The latter study also investigated **carbon monoxide** concentration and found a dose–response association with dementia risk for this exposure (adjusted HR compared to the lowest quartile, 95 % CI: 1.07, 0.92-1.25; 1.37, 1.19-1.58; 1.61, 1.39-1.85) [28]. Similar patterns were seen for men and women separately.

**Environmental tobacco smoke** was shown to be associated with dementia risk in a cross-sectional study of almost 6000 people in five provinces of China [29]. These investigators asked participants about exposure to environmental tobacco smoke at home, work, and in other locations, duration of exposure as well as estimating cumulative exposure. Rather than a clinical diagnosis, these investigators used the GMS-AGECAT algorithm which has been widely used in epidemiological studies, including the MRC Cognitive Function and Ageing Studies [30, 31]. Higher levels of exposure were associated with an increased risk of severe dementia (O3-5) but not moderate dementia (O1-2). The cut off for moderate dementia used in this study showed only a positive predictive value of 68.6 % but the cut off used for severe dementia performed much better (88.1 %) [32]. The cumulative dose analysis showed a dose–response association (adjusted HR compared to a cumulative dose of zero for groups >0-24, 25–49, 50–74, 75–99, ≥100, 95 % CI: 0.99, 0.76-1.28; 1.15, 0.93-1.42; 1.18, 0.87-1.59; 1.39, 1.03-1.84; 1.95, 1.34-2.83). Also, all effect sizes were larger in never smokers than when ex-smokers were included.

A prospective Taiwanese study following almost a million people over ten years found an association between baseline **ozone** but not particulate matter up to 2.5 μm in diameter (**PM**_**2.5**_) and incident Alzheimer’s dementia [33]. However, change in exposure to both pollutants during follow up was associated with a two-to-three-fold increased risk of incident AD. A final cross-sectional study of 871 people in Taiwan also examined both particulate matter (**PM**_**10**_) and **ozone** concentration at the participant’s home address, finding increased Alzheimer’s dementia risk in the second and third tertiles of PM_10_ concentrations (adjusted OR, 95 % CI: 1.68, 0.94-3.00; 4.17, 2.31-7.54; P_trend_ < 0.001) but only in the highest tertile of ozone concentrations (0.60, 0.33-1.09; 2.00, 1.14-3.50; P_trend_ = 0.03) [34]. They found similar patterns of association with vascular dementia.

Thus, the evidence for airborne environmental risk factors for dementia is consistent in the direction of association for all exposures and the overall strength of evidence, while based on relatively few studies, is moderate to strong.

### Toxic heavy metals

Table 4 summarises the studies linking toxic heavy metals to dementia risk. There were two studies of **arsenic**, one of which was of grade-B quality and found no association with dementia; the grade-C study found increased rates of dementia in areas with higher soil arsenic levels but this country-level simulation is less informative about risk factors in individuals [35, 36]. A case–control study with 129 people in each group found that an excess of people with Alzheimer’s dementia were born in areas with higher than average **lead** concentrations [37].

Overall, there was relatively little extant evidence for an association between toxic heavy metals and dementia risk.

### Other metals

Table 5 summarises the studies focused on other metals and dementia risk. **Aluminium** was the most studied metal, with sixteen studies investigating its relationship with dementia including almost 22,000 people with dementia. The only high quality study – a prospective cohort study of almost four thousand older adults in south-west France (the PAQUID study [38]) – found that levels of aluminium consumption in drinking water in excess of 0.1 mg per day were associated with a doubling of dementia risk and a three-fold increase in the risk of Alzheimer’s dementia [39]. Of the remaining thirteen moderate quality studies, six found an association between increased aluminium levels in drinking water and increased dementia risk [40–45], four found no association [37, 46–48], and one found a protective effect of higher soil levels of aluminium [49]. In general, the larger studies showed a positive association and the smaller studies showed a null effect.

A prospective cohort study compared **calcium** levels in tap water with risk of developing Alzheimer’s dementia. However, it is unclear if their not mentioning calcium as a predictor means that they failed to examine it or if it did not emerge from the models as an independent predictor. A cross-sectional study found no association between **cobalt** and dementia risk [37, 50]. Two case–control studies and one review article summarising 101 studies considered **copper** and **iron** in relation to dementia [24, 37, 49]. The findings regarding copper were inconclusive. However, they reported that higher soil levels of iron were associated with an increased risk of dementia [37, 49]. The review article was inconclusive [24].

An excess of people with Alzheimer’s dementia were born in areas with higher than average levels of **manganese** in a cross-sectional study [37]. The same study found no evidence for an association between **molybdenum**, **nickel**, **uranium**, or **zinc** with dementia risk [37]. However, another study found that higher zinc levels in the soil was associated with an increased risk of Alzheimer’s dementia [49].

It is challenging to synthesise the published reports on the association between metals and dementia, and the published evidence for an individual element is often weak and/or contradictory. However, there is at least some evidence that calcium, cobalt, molybdenum, nickel, and uranium are not associated with an increased risk of dementia, but this is often based on minimal evidence. One prospective study and the larger cross-sectional studies tend to support an association between aluminium and dementia risk [39–41, 44].

### Other trace elements

Table 6 shows the studies investigating links between other trace elements and dementia. A cross-sectional study extrapolated from the medical records of 160 people to estimate annual dementia incidence and found the area with the highest **fluoride** concentrations in public water supplies had the lowest incidence [51]. **Selenium** has been the focus of a number of studies. The included review article [23] referred to a randomised, controlled trial of selenium supplementation to reduce dementia risk, the PREADVISE trial, but this seems to have converted into an observational study and is yet to report on selenium levels [52–54]. The review also referred to a conference abstract reporting a prospective cohort study [55], but subsequent reports do not focus on selenium [56]. In general, this review article reported mixed findings from 15 case–control studies and 24 autopsy studies [23].

One cross-sectional study found no differences in average levels of **silicon** in drinking water between a group of people with Alzheimer’s dementia and several neurological control groups [46]. On the other hand, two prospective cohort studies found an association between higher levels of **silica** in drinking water and dementia incidence [39, 57]. However, a case–control study did not corroborate these findings [48].

The evidence for other trace elements is again mixed and generally weak in strength.

### Occupational-related exposures

Table 7 summarises the individual studies relating occupational exposures to dementia risk. Four studies investigated occupational exposure to **aluminium** in relation to dementia [58–61]. These tended to be small with consequently low statistical power. Their results were mixed. The prospective Manitoba Study of Health and Aging studied a variety of exposures but the only robust association they found was an increased risk of dementia in relation to self-reported exposure to **defoliants/fumigants** [62]. The same study reported null associations in all other exposures studied, apart from a protective effect of **excessive noise** and increased risk associated with **radiation** exposure. However, in both these cases, only one person with dementia had been exposed to noise or radiation and so there is very limited statistical power. A case-cohort study of 1552 people dying with non-vascular dementia found no association between exposure to **diesel motor exhaust fumes** and dementia but mixed evidence for **extremely low frequency magnetic fields** or **electric shocks** [63]. Two reviews concluded that exposure to **pesticides** was associated with an increased risk of dementia which was corroborated by the prospective Canadian Study of Health and Aging, Cache County Study, the PAQUID study, and to some extent a retrospective British study of farmers but not by the Manitoba study or a case–control study from Québec; a study from the Netherlands had mixed findings [62–70]. A systematic review found no support for an association between occupational exposure to **lead** and dementia and mixed evidence (predominantly null) between **solvent** exposure and dementia [68]. A case–control study from the USA found an association between solvent exposure and AD, at least in men [71]. However, a case-cohort study from the Netherlands found mixed associations between solvents and dementia, perhaps reflecting the diversity of chemicals described as solvents [63].

The evidence linking occupational exposures and dementia is frequently weak. Aside from the mixed evidence for solvents, the strongest evidence is for exposure to pesticides, but even findings in that literature are mixed. However, there seems to be no published evidence suggesting that occupational exposure to diesel motor exhaust, lead, inks/dyes, paints/stains/varnishes, gasoline/fuels/oils, liquid plastics/rubbers, or vibratory tools affect dementia risk.

### Miscellaneous environmental factors

Table 8 summarises studies linking miscellaneous environmental exposures and dementia. One study reported no association between **climate** and dementia admissions [72]. Two systematic reviews examined low and extremely low frequency **electric and magnetic fields** and, while the evidence is mixed, there seems to be an association with dementia risk and this was corroborated by a prospective study in Switzerland which found that living close to power lines for over 15 years was associated with a doubling of Alzheimer’s disease mortality (but not the occupational study mentioned above [63]) [73, 74]. Its findings are difficult to interpret, but a prospective study in Denmark found that **mobile phone** subscription was associated with a decreased risk of subsequent hospital admission with dementia [75]. Three high quality prospective studies (including 11,884 people of whom 691 developed dementia) examined the association between **vitamin D** and dementia and all found that lower vitamin D levels at baseline were associated with an increased risk of developing dementia [76–78]. However, this finding was not corroborated by a small case–control study (40 in each group) of people with mild dementia [79]. Finally, one study noted that the area with the highest dementia mortality had the lowest drinking water **pH** [42].

Out of the miscellaneous exposures considered in this section, there seems to be strong evidence that vitamin D deficiency is associated with increased risk of dementia and moderate evidence implicating electric and magnetic fields. There is no published evidence supporting any role for weather parameters in dementia risk.

## Discussion

### Main findings

In summary, we found moderate evidence for air pollution exposures being related to dementia risk, particularly nitrogen oxides, particulate matter, and ozone. There was little evidence that toxic heavy metals, or indeed most metals, influence dementia risk, apart from aluminium – where larger, better quality studies suggested an association. Other than silicon, there was little evidence for other trace elements affecting dementia risk, though selenium remains an interesting element. Of the occupational exposures, there was little strong evidence, but the evidence suggests that exposure to some pesticides and, possibly, metals may affect dementia risk. Finally, there was strong evidence for vitamin D deficiency being associated with raised dementia risk and moderate evidence for electromagnetic fields, though this complicated exposure requires some unpicking.

### Limitations of the review and risk of bias within and across studies

The broad search and systematic methodology of this review is likely to have identified all the available literature. Our exclusion of studies without dementia as their outcome resulted in the exclusion of high quality studies examining the association between environmental risk factors and, for example, cognition or brain structure [80, 81]. Such studies may shed some light on the pathogenesis of dementia since cognitive or brain changes are important features of dementia but they are not specific to this syndrome. We excluded papers measuring levels of a particular substance (often trace elements) in brain areas or in serum since these physiological changes could not be directly linked to environmental exposure. Our other exclusion criteria (case studies, animal or nutritional studies) are unlikely to have resulted in the exclusion of relevant papers or introduced bias. As mentioned above, we did exclude papers examining the effect of rurality or urbanicity since we have previously reviewed this literature and found in a meta-analysis an increased risk of dementia (particularly Alzheimer’s dementia) in rural areas, particularly living in rural areas early in life [16]. One unavoidable bias is that, despite the projected increase in dementia rates occurring disproportionately in low-to-middle income countries [4], the majority of the research was conducted in high-income countries.

One of the major challenges in trying to synthesise such a broad selection of papers is the diversity of methodologies used. When considering Bradford Hill’s criteria for inferring a causal association (strength, consistency, specificity, temporality, biological gradient, plausibility, and coherence), one clearly cannot satisfy the temporality criterion with a cross-sectional study, and thus a longitudinal study will provide more robust evidence for the role of a risk factor in the pathogenesis of dementia [82]. Since many of the included studies are cross-sectional, it is often not possible to infer a causal relationship. However, attrition in longitudinal studies can be non-random which can introduce selection bias [83]. Furthermore, no study measured exposures at more than one time point (though some measured the average exposure in a period), which will give a more accurate picture of true exposure over time and is essential in order to test whether there are critical or sensitive periods within the life course [84].

The fact that a number of exposures were only studied in a single study (see Table 2) also weakens support for them as it is impossible to examine the consistency of the association in multiple studies. However, exposures which were investigated in more than one study were also measured in a variety of ways, sometimes with variable quality. For example, many exposures were inferred from an address. This may be reasonable when it is not possible to measure an individual’s exposure directly, but the timing of measurement of the exposure is crucial, as will be discussed below. One strength of this approach is that it allows cumulative exposure to be estimated, given the participant’s residential history. A more robust measure of an individual’s exposure is direct measurement. This will clearly make a study more costly and will also not solve the problem of the timing of the exposure. Less robust, but frequently used for occupational exposures, in particular, is self-report. This does avoid the problem of temporality as different points in the life course can be covered, but will be limited in detail and prone to recall bias [85]. Finally, direct measurement of levels of a substance in the brain is likely to be the most accurate, but is also the most difficult to link to dementia risk – particularly for substances present in health – and thus these studies were excluded from the presence of the review. Alzheimer’s disease in the brain and the overt clinical symptoms of Alzheimer’s dementia are parallel, related phenomena [9] and the fact that approximately a third of people dying without dementia can have moderate or severe Alzheimer’s disease in their brains further complicates the interpretation of autopsy studies [86]. Returning to Bradford Hill’s criteria, a number of included studies grouped their exposures into multiple categories, allowing the exploration of a biological gradient in any association observed – for example nitrogen oxides [27] – which can strengthen any inference of a causal relationship.

Where possible, we reported the most-adjusted model in a particular study. Most studies adjusted for age and sex and some for other relevant known risk factors for dementia such as educational attainment or comorbidity but adjustment beyond that was highly variable. The possibility of residual confounding still remains.

There was a similar diversity of methodologies (and diagnostic criteria) used to identify people with dementia and it is important to consider whether any of these may introduce bias. Most robust is direct clinical assessment of individuals (often combined with a preliminary screening phase to minimise costs), either specifically for a research project or sourced from medical records. Two-phase screening seems to provide an accurate measure of dementia [87], but screening non-participation may introduce selection bias [88]. A substantial number of studies used dementia mortality as their outcome. This has previously been criticised [89], but more recent studies suggest that dementia reporting is improving, for example 72 % of a memory clinic cohort had dementia correctly diagnosed on their death certificates [90]. Importantly, death certification of dementia did not seem to be related to premorbid intelligence or area-level deprivation in that study (unpublished results available from the author on request). Another study using multiple sources from electronic health records to identify cases of dementia found that death certificates alone identified 83 % of the total number of cases identified by any source [17]. An additional important point is the adequacy of controls in case–control studies – there was some variability in the extent of matching with people with dementia.

Since we know that dementia is a condition which is affected by risk and protective factors throughout the life course [1], *when* the exposure is measured is crucial. Within life course epidemiology, there are three models of the association between a risk factor and an outcome: accumulation of risk, sensitive periods, and critical periods [91, 92]. Sensitive periods refer to a period of the life course when an exposure has a greater effect than other times. A critical period is the only time when a particular exposure has its effect; it does not affect risk if encountered at a different point in life. Crucially for dementia – which is now indisputably considered a condition of the life course [93] – the exposure in a study must be measured sufficiently early in life plausibly to be involved in the pathogenesis of a condition which begins years or even decades before the clinical onset of symptoms [94]. For example, we have found evidence above for an association between air pollution and dementia but it is not clear at what stage of life exposure is important. A recent study found a biomarker which has been proposed for Alzheimer’s disease (reduced cerebrospinal fluid levels of Aβ_1–42_ [95]) in children in Mexico City who had been exposed to high levels of air pollution in utero and throughout their life compared to controls [96]. The issue of timing of exposures has been rather neglected by the published literature on potential environmental risk factors for dementia and should be considered in much richer detail in the future.

### Comparison with previous literature

To the best of our knowledge, this is the first comprehensive systematic review of environmental risk factors for dementia. A review article was recently published on environmental risk factors for Parkinson’s disease and Alzheimer’s disease [20]. However, this article did not take the systematic approach or focus on dementia of the present review – the author included an extremely broad range of risk factors, including some environmental risk factors alongside clinical, lifestyle, and dietary factors. The findings of that review generally agreed with ours and it concluded that there was robust evidence for an increased risk of Alzheimer’s disease in relation to exposure to pesticides and that there was weak evidence for an increased risk in relation to levels of aluminium in drinking water and electromagnetic fields.

An older review summarised the literature relating to a number of risk and protective factors and concluded that there was evidence that occupational exposure to solvents, pesticides, and electromagnetic fields as well high levels of aluminium in drinking water were hazardous [21]. A brief review article from around the same time considered young-onset Alzheimer’s dementia and highlighted a number of deficiencies in the literature, many of which remain, which frequently consisted of small studies with limited measurements of environmental data, particularly in relation to the life course and what would now be referred to as the ‘preclinical’ period [97].

### Implications

As mentioned above, if a modifiable environmental risk factor for dementia could be identified and modified, the implications for public health, the economic cost of dementia, and individuals’ suffering could be profound. We have reported, albeit with many caveats, a short list of environmental factors which may be related to dementia risk – air pollution (all types); aluminium; silicon; selenium; pesticides; vitamin D; and electric and magnetic fields – and it is reasonable to speculate about possible mechanisms which might underlie these associations, if they were to prove causal. These mechanisms could be examined in more detail using methodologies such as Mendelian randomization [98].

Air pollution has been shown to be associated with reduced cerebral blood flow [99] and seems to be neurotoxic [100]. As shown above, brain changes – including cognitive changes and biomarkers – can be identified in children living in areas with high levels of air pollution compared to controls [96]. Aluminium has been found in amyloid plaques and neurofibrillary tangles (pathological features of Alzheimer’s disease) and, in rat models, aluminium intake increases amyloid expression [101, 102]. Furthermore, it has been suggested that relatively small amounts of aluminium could be neurotoxic and levels could accumulate selectively in certain brain tissues [103]. Silica may reduce absorption or increase excretion of aluminium [39]. Copper could be implicated in the pathogenesis of Alzheimer’s disease in a number of ways, including promoting Aβ aggregation and hyperphosphorylation and aggregation of tau [104]. The role of selenium in human physiology is complex [105] and it is involved in multiple molecular pathways relevant to the development of Alzheimer’s disease [23]. Pesticides form a heterogeneous group but some seem to be neurotoxic. Organophosphates, for example, may disrupt cholinergic neurotransmission through inhibition of acetylcholinesterase [69]. Chronic exposure to some pesticides has been linked to multiple neurological conditions, though specific mechanisms remain unclear. Vitamin D has numerous effects relevant to the pathogenesis of Alzheimer’s disease, including stimulating macrophages to clear amyloid plaques, reducing amyloid-induced cytotoxicity, as well as maintenance of cerebrovascular function [78]. Electric and magnetic fields have been proposed to affect the brain in a variety of mechanisms – including oxidative stress, apoptosis and necrosis of neurons, and even cytogenetic effects – but no firm mechanistic link with dementia has been made [73, 106]. It can be seen that several of these putative risk factors could act through multiple routes and it may become even more difficult to disentangle the relative importance of Alzheimer’s disease pathology and cerebrovascular disease in causing clinical dementia.

## Conclusions

In conclusion, the published evidence concerning specific environmental risk factors for dementia is generally not strong. However, there seems to be little role for most metals or other trace elements, occupational exposure to lead, inks/dyes, paints/stains/varnishes, gasoline/fuels/oils, liquid plastics/rubbers, vibratory tools, or climate in determining dementia risk. There is at least moderate evidence consistently supporting air pollution, aluminium, silicon, selenium, pesticides, vitamin D, and electromagnetic fields as putative environmental risk factors for dementia. More and better research is needed and we suggest that this shortlist should form the initial focus of attention.

## Appendix 1

Search syntax for PubMed and Web of Science

Alzheimer’s OR dementia AND

1. air pollution (air pollut*)
2. climate
3. UV radiation
4. green space
5. industrial pollution (indust* pollut*)
6. drinking water
7. noise pollution
8. low-frequency radiation
9. radiofrequency radiation
10. radon
11. nuclear facilities (nuclear facil* OR nuclear power)
12. contamination (contaminat*)
13. trace elements
